# Vaginal repair with mesh versus colporrhaphy for prolapse: a randomised controlled trial

**DOI:** 10.1111/j.1471-0528.2009.02254.x

**Published:** 2009-07-07

**Authors:** M Carey, P Higgs, J Goh, J Lim, A Leong, H Krause, A Cornish

**Affiliations:** Department of Urogynaecology, Royal Women’s Hospital, Melbourne, Vic.Australia

**Keywords:** Colporrhaphy, mesh, pelvic organ prolapse, randomised controlled trial

## Abstract

**Objective:**

To compare vaginal repair augmented by mesh with traditional colporrhaphy for the treatment of pelvic organ prolapse.

**Design:**

Prospective randomised controlled trial.

**Setting:**

Tertiary teaching hospital.

**Population:**

One hundred and thirty-nine women with stage ≥2 prolapse according to the pelvic organ prolapse quantification (POP-Q) system requiring both anterior and posterior compartment repair.

**Methods:**

Subjects were randomised to anterior and posterior vaginal repair with mesh augmentation (mesh group, *n*= 69) or traditional anterior and posterior colporrhaphy (no mesh group, *n*= 70).

**Main outcome measures:**

The primary outcome was the absence of POP-Q stage ≥2 prolapse at 12 months. Secondary outcomes were symptoms, quality-of-life outcomes and satisfaction with surgery. Complications were also reported.

**Results:**

For subjects attending the 12-month review, success in the mesh group was 81.0% (51 of 63 subjects) compared with 65.6% (40/61) in the no mesh group and was not significantly different (*P*-value = 0.07). A high level of satisfaction with surgery and improvements in symptoms and quality-of-life data were observed at 12 months compared to baseline in both groups, but there was no significant difference in these outcomes between the two groups. Vaginal mesh exposure occurred in four women in the mesh group (5.6%). De novo dyspareunia was reported by five of 30 (16.7%) sexually active women in the mesh group and five of 33 (15.2%) in the no mesh group at 12 months.

**Conclusion:**

In this study, vaginal surgery augmented by mesh did not result in significantly less recurrent prolapse than traditional colporrhaphy 12 months following surgery.

## Introduction

In the United States, 200 000 women undergo surgery annually for pelvic organ prolapse.^1^,^2^ Combined anterior and posterior colporrhaphy was performed on 35.2% women undergoing prolapse surgery in 2003 and was the most common operation for this condition.^2^ A lifetime risk of 11.1% for surgery to treat pelvic organ prolapse or urinary incontinence or both was reported by a study from a United States health maintenance organisation.^3^ Within 4 years of the primary surgical procedure, further surgery for recurrent prolapse and/or incontinence was required in 29.2%.^3^

Dissatisfaction with traditional colporrhaphy for pelvic organ prolapse has resulted in increased use of mesh to augment vaginal repair procedures to obtain higher success rates. However, the use of mesh during vaginal repair procedures is controversial. Uncontrolled studies have reported significant problems (e.g. dyspareunia and mesh exposure) with the use of mesh during vaginal prolapse surgery.^4^,^5^ By contrast, there is wide acceptance of mesh use for prolapse with the abdominal sacral colpopexy procedure.^6^–^8^

This study was designed to evaluate whether vaginal surgery with mesh augmentation would reduce the rate of recurrent prolapse at 12 months when compared with traditional colporrhaphy. We also evaluated complications, symptoms, quality-of-life outcomes and patient satisfaction with surgery.

## Methods

Women recommended vaginal surgery for anterior and posterior vaginal wall prolapse with stage 2 or more prolapse according to the pelvic-organ-prolapse quantification (POP-Q) system were invited to participate in this study.^9^ Women requiring only anterior or posterior compartment repair or with prolapse of the vaginal vault or cervix beyond the hymen or, in the opinion of the assessing surgeon, required abdominal prolapse surgery with mesh (e.g. open or laparoscopic sacral colpopexy) were excluded from the study. Women with contraindications for mesh usage, such as prior pelvic radiotherapy, pelvic sepsis, planned future pregnancy or immunocompromised were ineligible for the study. Institutional research and ethics committee approval for this study was obtained. All eligible women who agreed to participate in this study and provided written informed consent were enrolled between February 2003 and August 2005.

A sample size of 128 women (64 in each group) was required to achieve a significance level of 0.05 with a power of 0.8. This was based on the assumptions of 71% cure for traditional colporrhaphy and 93% for vaginal repair with mesh augmentation and a 15% loss to follow-up rate.^3^,^10^ Randomisation was computer generated and assignment was revealed prior to surgery. Subjects and surgeons were not blinded to the intervention.

Patients were assessed clinically and the prolapse staged using the POP-Q quantification system. Multichannel urodynamics was performed on subjects with urinary incontinence prior to surgery. All subjects completed validated questionnaires prior to surgery and at 6 and 12 months following surgery. The questionnaires were the Prolapse Symptom Inventory and Quality of Life questionnaire (PSI-QOL), Short-form Urogenital Distress inventory (SUDI), Short-form Incontinence Impact questionnaire (SIIQ) and Cleveland Clinic Continence score (CCCS).^11^–^13^ The subjects also completed a visual analogue scale (VAS; 0-100 where 100 represented being completely satisfied and 0 completely dissatisfied) of their satisfaction with surgery at 6 and 12 months following surgery.

The primary outcome was objective success of surgery as defined by the absence of POP-Q stage 2 or more prolapse (i.e. no prolapse at or below a point 1 cm above the hymen at any vaginal site) 12 months following surgery. Secondary outcomes were symptoms, quality-of-life outcomes and patient satisfaction with surgery at 6 and 12 months. Complications of surgery were also reported.

If a tension-free vaginal tape (TVT) or trans-obturator tape was required, this was undertaken at the start of surgery. A vaginal hysterectomy was then performed for women requiring this procedure. The vagina repair procedure was then performed. A perineal repair was performed as required.

Patients randomised to the no mesh group underwent standard anterior and posterior colporrhaphy. Plication of the pre-vesical and pre-rectal tissue with 2/0 polydioxanone sutures was performed. Excess vaginal epithelium was excised as required and closed with a continuous locking suture.

**Figure 1:**
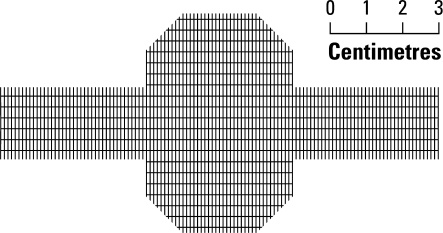
A cross-shaped mesh was used for the anterior vaginal repair. The extension arms were placed into each paravaginal space.

When mesh was used in the anterior vaginal repair, a full thickness midline epithelial incision was made. The vaginal epithelium was mobilised off the underlying pre-vesical tissue. Dissection continued towards each arcus tendineus fascae pelvis (ATFP). The inner aspect of the pubic bone was palpated at the level of the mid-vagina and lateral dissection was then continued through the ATFP with fine scissors using a ‘push-spread’ technique for approximately 3 cm. Only the central area of the pre-vesical fascia was repaired with 2/0 Monocryl (Ethicon, Somerville, NJ, USA). This avoided narrowing the pre-vesical space. The mesh (Gynemesh PS; Ethicon) was soaked in an antibiotic solution prior to placement and liberal wound irrigation with saline was performed during surgery. A cross-shaped piece of mesh was cut and placed over the pre-vesical tissue with the extension arms placed into each paravaginal space (Figure 1). The mesh extension arms abutted the inner aspect of the pubic bone on each side. Excess vaginal epithelium was trimmed as required and closed with a nonlocking continuous everting mattress suture.

**Figure 2:**
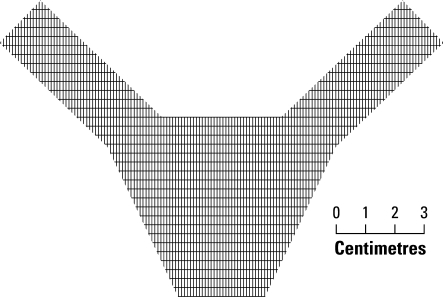
A ‘Y’-shaped mesh was used for the posterior vaginal repair. The extensions arms abutted each sacrospinous ligament.

When mesh was used to reinforce the posterior vaginal repair, a full thickness midline epithelial incision was made. The vaginal epithelium was dissected off the underlying pre-rectal tissue. Dissection continued laterally on each side to the levator ani muscles. At the apex, dissection continued through the rectal pillars to each ischial spine and sacrospinous ligament. Only the central area of the pre-vesical fascia was repaired with 2/0 Monocryl. This prevented narrowing the pre-rectal space. A ‘Y’-shaped piece of mesh was cut and placed over the pre-rectal tissue with the extension arms placed in the tunnels created by the dissection onto the sacrospinous ligaments (Figure 2). The mesh extension arms abutted each sacrospinous ligament. Mesh was not placed in the lower third of the posterior vaginal wall. The vaginal epithelium was trimmed as required and closed with a nonlocking continuous everting mattress suture.

Sacrospinous ligament fixation of the vaginal vault or uterus as described by Carey and Slack was performed at the discretion of the surgeon.^14^ For seven subjects, laparoscopic suture sacral hysteropexy was performed as described by Krause *et al.*^15^

All patients received intra-operative antibiotics and thromboprophylaxsis. For subjects assigned to the mesh group, perioperative intravenous antibiotics were continued for 48 hours followed by oral antibiotics for a further 5 days.

Follow-up examination was performed at 6 and 12 months. During the examination, the examiner attempted to remain blinded to the surgical intervention received by each subject. Patient satisfaction using a visual analogue score (VAS) and validated symptom and quality-of-life questionnaires (Prolapse Symptom Inventory & QOL [PSI-QOL]) were administered at 6 and 12 months postoperatively.

Fisher’s exact test was used for discrete outcomes. Two-sample *t*-test was used for parametric continuous data.

## Results

**Figure 3:**
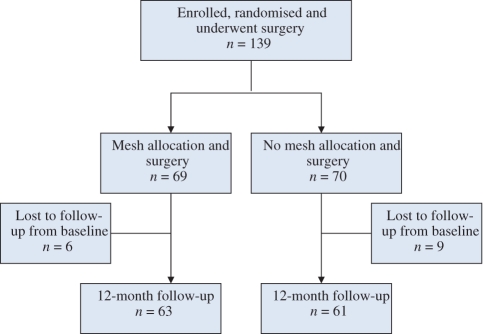
Study flow chart.

We recruited 139 women into the study (Figure 3). Departure from study protocol occurred in seven women with uterine prolapse who underwent laparoscopic suture sacral hysteropexy (two in the mesh and five in the no mesh group), two women who underwent a single compartment posterior repair (one in the mesh and one in the no mesh group) and one subject treated without mesh with a stage 4 uterine prolapse. In an attempt to replace these subjects, a further 11 women were recruited into the study in addition to the 128 planned recruits. All 139 women remained in the final analysis based on an intention-to-treat analysis. Therefore, for purposes of analysis, there were 69 women in the mesh group and 70 women in the no mesh group. There were no significant differences in demographics between the two groups (Table 1).

**Table 1:** Demographics and preoperative details for mesh and no mesh groups

Variable	Mesh (*n*= 69)	No mesh (*n*= 70)	*P*-value
Age in years, mean (SD)	59.1 (±11.4)	57.6(±11.0)	0.42*
Parity, mean (SD)	3.24 (±1.59)	3.42 (±1.62)	0.51*
BMI (kg/m^2^), mean (SD)	28.89 (±5.56)	28.66 (±5.04)	0.81*
No. subjects analysed^1^	63	61	
Hormone therapy	18.5%	15.7%	0.80**
No. subjects analysed^1^	54	51	
Prior Prolapse Surgery	13.6%	26.2%	0.08**
Prior Hysterectomy	31.9%	41.4%	0.29**
No. subjects analysed^1^	22/69	29/70	
Dyspareunia^5^	32.4%	55.6%	0.06**
Proportion^1^	11/35	20/34	

* Two sample *t*-test.

** Fisher’s exact test.

Of the 139 women recruited, 108 (58 in the mesh group and 50 in the no mesh group) attended for the 6-month follow up and 124 (63 in the mesh group and 61 in the no mesh group) attended for the 12 month follow up. We chose to report the 12-month results for the primary and secondary outcomes.

A mid-urethral sling for stress incontinence was performed in 49% of the mesh group and 33% of the no mesh group. Twenty-seven percent of women in the mesh group and 28% in the no mesh group had a vaginal hysterectomy. Sacrospinous fixation was performed in 58% of the mesh group and 47% in the no mesh group.

The primary and secondary outcomes at 12 months are detailed in Table 2. For the primary outcome, we examined varying assumptions about data from women lost to follow up. Results are reported just for women returning for follow up (i.e. assuming that data were missing at random); assuming that women lost to follow up were treatment failures; and assuming that women lost to follow up were treatment successes.

**Table 2:** Primary and secondary outcomes at 12 months following surgery

Variable	Mesh (*n*= 69)	No mesh (*n*= 70)	*P*-value
Objective success (POP-Q stage 0 or 1)at 12 months of subjects returning forfollow up	51/63 (81.0%)	40/61 (65.6%)	0.07*
Objective success (POP-Q stage 0 or 1)at 12 months assuming subjects lost tofollow up as failures	51/69 (73.9%)	40/70 (57.1%)	0.049*
Objective success (POP-Q stage 0 or 1)at 12 months assuming subjects lost tofollow up as successes	57/69 (82.6%)	49/70 (70.0%)	0.11*
PSI-QOL, mean change from preoperativeto 12 months score** (SD)	−6.93 (±8.25)	−7.77 (±7.43)	0.58***
No. subjects analysed	55	53	
SUDI, mean change from preoperativeto 12 months score** (SD)	−20.4 (±29.5)	−17.6 (±30.9)	0.62***
No. subjects analysed	59	57	
SIIQ, mean change from preoperative to12 months score** (SD)	−17.3 (±30.9)	−15.0 (±33.2)	0.76***
No. subjects analysed	35	37	
CCCS, mean change from preoperativeto 12 months score** (SD)	−1.00 (±4.20)	−0.75 (±4.30)	0.78***
No. subjects analysed	50	44	
Subjects reporting a VAS for satisfactionwith surgery of ≥80/100	45/59 (91.5%)	51/63 (81.0%)	0.12*
Awareness of prolapse	3/61 (4.9%)	7/62 (11.3%)	0.32*

* Fisher’s exact test.

** A negative change indicates a decrease in score over time and improved symptoms or impaired quality of life.

*** Two sample *t*-test for independent means.

Recurrent prolapse occurred most commonly in the anterior compartment. In the mesh group, no recurrence was more than point 0 and in the no mesh group, no recurrence was more than point +1 on POP-Q examination. Of those women with recurrences, one woman has undergone a laparoscopic sacral colpopexy and another has undergone a vaginal repair with mesh. Both subjects were from the no mesh group.

The results of PSI-QOL, SUDI, SIIQ and CCCS questionnaires are detailed in Table 2. For all four questionnaires, higher scores indicate worsening symptoms or impaired quality of life. The changes in scores for each scale from baseline to 12 months following surgery have been reported. Therefore, a positive change indicates an increase in score over time and a negative change indicates a decrease in score over time. For all scales, a negative change in score was observed from baseline to 12 month after surgery indicating improved symptoms or quality of life.

Intra-operative complications included one bladder perforation and one bowel perforation in the no mesh group. Both perforations were noted during the surgery and repaired intra-operatively without postoperative sequeale. One subject in the mesh group experienced significant intra-operative blood loss.

Postoperative complications included four cases (5.6%) of vaginal mesh exposure in the mesh group. Three mesh exposures were anterior and one was both anterior and posterior. Only one patient who developed a mesh exposure had a concomitant vaginal hysterectomy. Three of the mesh exposures presented at the 6-week postoperative review and one presented at 12 months. Three mesh exposures were treated surgically by simple excision of the exposed mesh and one case was managed conservatively. There was one tape exposure from a TVT procedure in the no mesh group.

Women were questioned regarding sexual activity and dyspareunia preoperatively and at 6 and 12 months. Eleven of 34 (32.4%) sexually active women in the mesh group and 20 of 36 (55.6%) in the no mesh group described preoperative dyspareunia. This difference was not significant (*P*=0.07). At the 6-month review, six of 25 (24.0%) in the mesh group and 11 of 27 (40.7%) in the no mesh group reported dyspareunia (*P*=0.25). At 12 months, 12 of 30 (40.0%) in the mesh group and 13 of 33 (39.4%) in the no mesh group reported dyspareunia (*P*=1). De novo dyspareunia was reported by five of 18 (27.8%) sexually active women without preoperative dyspareunia in the mesh group and five of 12 (41.7%) in the no mesh group at 12 months (*P*=0.46). Dyspareunia following surgery was considered to be because of vaginal stenosis in three women in the mesh group and five women in the no mesh group. Two women underwent vaginoplasty for vaginal stenosis and both were from the no mesh group.

## Discussion

Our results failed to demonstrate that vaginal repair surgery augmented by mesh was significantly more successful in terms of reduced recurrent prolapse than traditional colporrhaphy 12 months following surgery. This finding was based on analysis of only those women who returned for the 12-month review. Alternative assumptions about missing data suggested a significantly higher success rate for the primary outcome measure for the mesh group when subjects not returning for review were assumed to be treatment failures.

While designing the study, we chose a relatively homogenous group of women requiring both anterior and posterior vaginal repair surgery with limited apical prolapse. This avoided the need for within group analysis and the problem of how to deal with women developing prolapse in the nonrepaired compartment.^16^ Departures from the study protocol were included in the final analysis on an intention-to-treat basis. However, the requirement for additional surgery for stress urinary incontinence and vaginal vault prolapse adds heterogeneity to the study population, and this together with protocol departures and missing data, undoubtedly effects the power of our study. Our failure to detect a difference between these procedures does not mean that such a difference could not exist.

Combined anterior and posterior colporrhaphy was chosen as the comparator as this is the most common procedure performed for prolapse in the USA.^2^ This operation was performed on 35.2% of 199 698 women undergoing prolapse surgery in the USA in 2003 compared to 17.0% of women undergoing anterior and 16.4% undergoing posterior colporrhaphy.^2^ Four randomised controlled studies have compared traditional colporrhaphy with vaginal repair with synthetic or biological graft augmentation with conflicting results. A recent study demonstrated that anterior colporrhaphy reinforced with mesh significantly reduced recurrent cystocele from 38.5 to 6.7% when compared with traditional anterior colporrhaphy.^17^ Another study reported that vaginal repair augmented by polyglactin 910 absorbable mesh significantly reduced recurrent cystocele from 43% in the no mesh group to 25% in the mesh group, but there was no difference in the rate of recurrent rectocele between the two groups.^18^ A further study demonstrated no significant difference in cystocele recurrence rates when three anterior repair techniques were compared, including one group with polyglactin 910 mesh reinforcement.^19^ Another study reported anterior colporrhaphy augmented by solvent dehydrated fascia lata did not reduce recurrent cystocele compared with traditional colporrhaphy.^20^ Two further studies have compared abdominal sacral colpopexy with transvaginal sacrospinous colpopexy.^8^,^21^ Both studies reported a trend towards the abdominal sacral colpopexy being associated with less recurrent prolapse and dyspareunia than sacrospinous colpopexy. Both studies have been widely interpreted as comparisons between abdominal and vaginal surgery for prolapse. However, in both studies, the subjects were randomised to prolapse surgery with mesh (abdominal sacral colpopexy) and without mesh (sacrospinous colpopexy) with less recurrent prolapse and dyspareunia reported in the mesh group.

The impact of surgery on sexual function is difficult to quantify. On direct questioning, the rates of dyspareunia were high in both groups preoperatively and at 6 and 12 months following surgery. These rates seemed to fluctuate with time. The high prevalence of dyspareunia is consistent with other studies.^22^,^23^ There was an improvement in sexual function according to the PSI-QOL questionnaire in both groups following surgery. Interestingly, some women who reported no sexual activity to the medical staff reported sexual dysfunction because of pelvic symptoms in the self-administered PSI-QOL questionnaire. This may reflect a reluctance to discuss these issues with the medical staff. We observed that de novo dyspareunia at 12 months following surgery was higher in the no mesh group compared with the mesh group. This may be explained by the different surgical techniques between traditional colporrhaphy and mesh-augmented repair. With colporrhaphy, the more lateral plication of the pre-vesical and pre-rectal tissues may result in reduced vaginal capacity compared with the mesh repair with plication of only the central pre-vesical and pre-rectal tissues.

The prevalence of vaginal mesh exposure (5.6%) in the mesh group is similar to rates reported for abdominal sacral colpopexy. A comprehensive review of abdominal sacral colpopexy by Nygaard *et al.* identified a 3.4% prevalence of mesh erosion.^6^ A more recent study of 313 women treated by abdominal sacral colpopexy reported a mesh erosion rate of 5.4%.^24^

## Conclusion

Our study showed no significant reduction in recurrent prolapse 12 months following anterior and posterior vaginal repair with mesh augmentation compared with standard anterior and posterior colporrhaphy. Given our sample size and the number of patients failing to attend follow up, conclusions regarding the primary outcome were sensitive to assumptions made regarding those lost to follow up. A larger study is required to more conclusively assess the effectiveness and safety of mesh-augmented vaginal repair surgery.
